# The TG/HDL-c Lipid Ratio as a Cardiovascular Risk Marker in a Mexican Urban Middle-Class Population: Do We Need a Risk Score Tailored for Mexicans?

**DOI:** 10.3390/jcm12186005

**Published:** 2023-09-16

**Authors:** Yolanda Martínez-Marroquín, Alejandra Meaney, Virginia Samaniego-Méndez, Nayelli Nájera, Guillermo Ceballos, Carlos Fernández-Barros, Eduardo Meaney

**Affiliations:** 1 Epidemiology Unit, Hospital Regional Toluca, ISSEMYM, Toluca 50010, Mexico; 2 Cardiovascular Unit, Hospital Regional “1° de Octubre”, ISSSTE, Lindavista, Mexico City 07760, Mexico; 3 Laboratorio de Investigación Cardiometabólica Integral, Sección de Estudios de Posgrado e Investigación, Escuela Superior de Medicina, Instituto Politécnico Nacional, Mexico City 11340, Mexico; nnajerag@ipn.mx (N.N.); gceballosr@ipn.mx (G.C.); 4 Hospital Ángeles, Torreón 27250, Mexico; carlosfernandezbarros@gmail.com

**Keywords:** risk scales, cardiovascular risk categories, Lindavista study, TG/HDL-c ratio

## Abstract

Introduction: Risk scores are essential in primary prevention to detect high-risk patients. The most common scores exclude hypertriglyceridemia and abdominal obesity in their risk assessment. We examined the triglyceride/HDL-cholesterol (TG/HDL-c) ratio as a cardiovascular (CV) risk marker in a middle-class urban Mexican population sample. Aim: Our aim was to test the concept of a scoring system reflecting Mexican population characteristics. Methods: A total of 2602 healthy adults from the Lindavista primary prevention program were considered, evaluating gender, age, blood pressure, smoking, body mass index, waist circumference, lipid profile, and fasting glucose. According to the abnormality, a score from −3 to +3 was assigned. Results: The summation of eleven variables yielded the Lindavista score (LS), which was calibrated versus the TG/HDL ratio and ACC ASCVD Risk Estimator Plus score to determine its correlation with risk categories. The TG/HDL-c ratio had a linear correlation with LS and high-risk ACC ASCVD categories. Conclusions: Compared with LS and TG/HDL-c, the ACC ASCVD system underestimates the high-risk category. The high prevalence of obesity and lipid triad in the Mexican population requires a scale that considers those traits. The TG/HDL-c ratio is a practical, easy, and economical instrument to categorize risk in Mexicans.

## 1. Introduction

Cardiovascular diseases (CVDs) are the leading cause of worldwide mortality [1]. As epidemiological transition affects more countries with medium and low incomes, the diseases rooted in extreme poverty, malnutrition, unhealthiness, and unavailable fresh water have decreased in global importance as causes of death. At the same time, the incidence, prevalence, and lethality of chronic degenerative diseases have increased [2]. Although the mortality from atherosclerotic coronary artery disease has fallen in numerous industrialized countries and some Latin American nations, it has increased significantly in other middle-developed countries, such as Mexico [3,4]. Due to transformations of all kinds—demographic, nutritional, socioeconomic, political, and psycho-cultural—that occurred in Mexico mainly in the second half of the last century, the epidemiologic profile of its population has changed drastically. Nowadays, Mexico is devastated by an epidemic of abdominal obesity, which, in turn, brought a paramount increase in the prevalence and mortality of type 2 diabetes mellitus (DM2). At the same time, high blood pressure (HBP), dyslipidemia, and ischemic heart disease (currently, the main cause of general mortality) [4] are growing epidemiological problems in Mexico [5,6,7]. In this regard, several governmental and academic-based epidemiological studies [6,7,8,9,10,11,12,13] have shown an increment of almost every major CV risk factor in Mexico, shaping the profile of a population threatened by a wave of coronary and cerebrovascular diseases in the present and the foreseeable future. Current evidence reveals that HBP affects 30% of adult Mexicans; obesity and overweight are found in 38.3% and 36.9% of the total population (75.2% of unhealthy weight), while abdominal obesity prevails in 81% of adults. Meanwhile, the prevalence of diabetes reached 18.3% of adults, and some form of dyslipidemia affects half of the population [14,15,16]. In the primary prevention of atherosclerotic cardiovascular diseases (ASCVDs), a precise system is indispensable for estimating CV risk and predicting coronary and cerebrovascular outcomes. Estimating risk helps to identify those individuals with a high probability of suffering coronary, cerebrovascular, or peripheral vascular morbidities and mortality, as well as heart failure, guiding and segmenting the application of preventive and therapeutic measures. All CVD risk scoring systems that are currently in use are based on multivariable regression equations derived from the data of specific single or pooled cohorts [17,18]. As these prospective cohort studies are expensive, complicated, and time-consuming, they came from developed, industrialized nations such as the United States and the European Union countries [18]. The United States of America’s population, even though it is composed of different ethnic groups, tends to remain unmixed (Afro-Americans and Whites), is experiencing slow miscegenation, which includes Latin American, Oriental, Arab, and Jewish ethnicities, among others. Even so, the American College of Cardiology atherosclerotic cardiovascular disease scale does not consider the US population of Latin American origin, which currently comprises about 62 million people (19% of the total population). As less developed nations do not have similar score-making studies, their physicians are forced out of necessity to use scores or markers for populations that are very different from their own from ethnic, anthropometric, nutritional, and cultural points of view [19].

Conversely, classical or novel biomarkers have been added to several multivariate risk systems to enhance their prediction value. Since the first Framingham observations, some very simple ratios among various serum lipid fractions have been used alone as independent risk markers [20,21,22,23,24]. This study is aimed to test the value of a well-known index, the ratio between serum triglycerides and cholesterol linked to high-density lipoproteins (TG/HDL-c), as a marker of ASCVD risk in a sample of a middle-class mestizo, urban Mexican population [13]. A second purpose was to test the concept of a scoring system based on some very prevalent anthropometric and metabolic traits in Mexican inhabitants which need to be considered in the current risk assessment to analyze our population correctly.

## 2. Materials and Methods

The subjects of the present study belong to the cohort of the Lindavista trial [13], a primary prevention multiple-intervention study focused on reducing CV risk factors in a non-probabilistic sample of Mestizo urban middle-class inhabitants of Mexico City who were recruited by invitation. The cohort comprised two randomized parallel arms, one where cardiologists performed therapeutic interventions and another where general practitioners conducted those procedures. The cohort was composed of 2602 patients of both genders, aged 35 or over, who did not know they had DM2 and without clinical evidence of cerebrovascular, cardiac, or peripheral arterial diseases or other relevant systemic disease at the time of enrollment. The protocol was approved by the institutional committees of ethics and investigation and conducted according to the guidelines of Good Clinical Practices [25], the standards from the Declaration of Helsinki [26], and the Federal regulations derived from the General Law of Health, which is part of the Mexican Constitution [27]. The studied population’s characteristics and the trial’s methodology have already been published [13]. Smoking status (current and past) was considered. Anthropometric data included weight, height, body mass index (weight in kg/squared size in cm), and abdominal circumference (cm). Systemic blood pressure (systolic and diastolic) was measured in mm of Hg, with a mercurial sphygmomanometer, following standard recommendations [28]. The metabolic assessment included fasting serum glycemia, total cholesterol (TC), triglycerides (TG), and HDL cholesterol (HDL-c). All measurements were determined by colorimetric assay kits and obtained in mg/dL. Low-density lipoprotein cholesterol (LDL-c) was estimated using the Friedewald formula [29] if concentrations of TG were below 300 mg/dL. If not, instead of LDL-c, the non-HDL cholesterol estimation was used as a substitute. Finally, the atherogenic ratio TG/HDL-c was calculated from the lipid values.

Eleven variables were considered (see Table 1), the sum of which was named the Lindavista score (LS). Arbitrary values between 0 and 3 (positive or negative) were assigned to each factor, according to their magnitude, considering the contemporary concepts and criteria of “normality” and pathologic deviation of each one. Therefore, the highest possible value of the risk score is thirty-three. Finally, for every patient, a total score was estimated, the magnitude of which would reflect the ASCVD accumulated risk. The LS of all the cohort participants was employed in the present analysis.

Furthermore, the CV risk in the cohort’s patients was assessed utilizing the ACC ASCVD Risk Estimator Plus score (ASCVD REP) [30,31], based on the United States-generated pooled cohort equations, which estimates a 10-year absolute risk for ASCVD, such as coronary death, nonfatal myocardial infarction, and fatal or nonfatal stroke. The equations consider age, gender, and ethnicity; systolic and diastolic blood pressures; TC, LDL-c; HDL-c; history of diabetes; smoking status; and current use of antihypertensive drugs, statins, and aspirin [27,28]. The score defines a low absolute risk if the value is <5%, borderline between 5% and <7.4%, intermediate if the score goes from 7.5% to <19.9%, and high risk when the score value is >20%.

Firstly, LS summations were distributed into quartiles and compared with the values of the TG/HDL-c values. Afterward, quartile values of LS summation were correlated against the ASCVD REP score, comparing the four classical ACC/AHA categories of low, borderline, intermediated, and high risks. Finally, the correlation between the ASCVD REP score and the TG/HDL-c index was determined using the quotient values corresponding to the quartile distribution of the LS sums.

### Statistical Analysis

Statistical analysis and quartile calculation were conducted using GraphPad Prism version 9.5.1 for Mac (GraphPad Software Inc., San Diego, CA, USA). The correlation coefficients among the ASCVD REP and Lindavista risks were performed using Pearson’s correlation coefficient formula. A *p*-value < 0.05 was considered statistically significant.

## 3. Results

Table 1 shows all the risk factors considered and the grading assignment. The quartile distribution showed that Q1 encompasses scores from 0 to 4.9; Q2, from 5 to 8.9; Q3, from 9 to 13; and Q4, greater than 13 scores.

**Figure 1:**
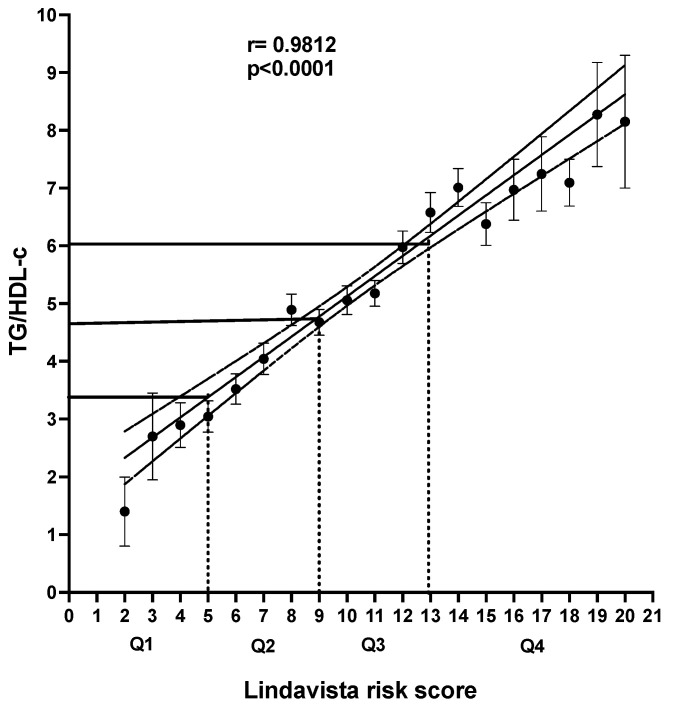
Calibration of the Lindavista score, correlating it with the TG/HDL index. The TG/HDL ratio values were compared with the Lindavista score values divided into quartiles to calibrate the meaning of LS sums. Dotted perpendicular lines represent the quartile values of LS. Meanwhile, the solid horizontal lines express the correspondence between LS estimations and the TG/HDL-c index values. The TG/HDL-c index figures < 3.3, 3.3–4.6, 4.7–6, and >6 correspond to each quartile distribution. It is remarkable the straight linear correlation between both variables.

Figure 1 shows the LS’s quartiles calibration as its values were compared to the index TG/HDL-c. There is a straight linear correlation, with a correlation coefficient > 0.98, between the two variables. The corresponding values of the TG/HDL-c index were less than 3.3 for Q1, in a range from 3.4 to 4.6 for Q2, in a band from 4.7 to 6 for Q3, and values > 6 for Q4.

**Figure 2:**
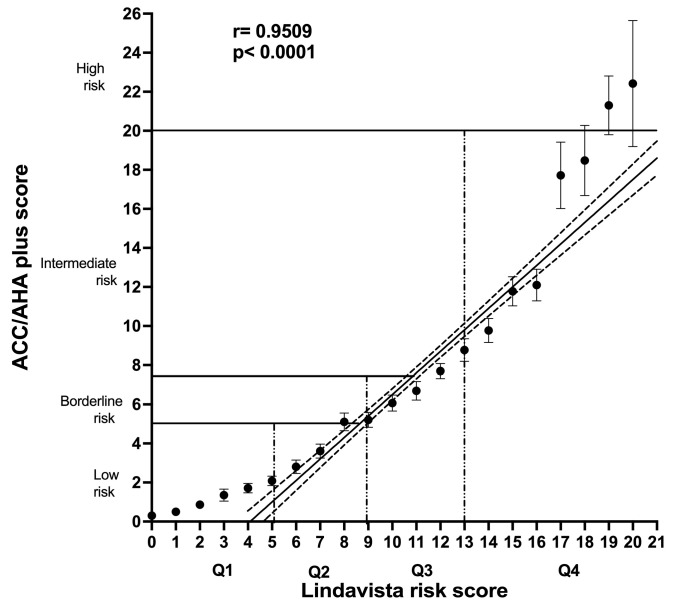
Calibration of the Lindavista score, correlating it with the ASCVD REP score. The correlation between ASCVD REP and quartiles of LS scores is also linear. Both scales coincide with low and borderline risk categories, but the US score system underestimates the LS intermediate and high risk. Most summations corresponding to upper-quartile LS summation correspond to intermediate ACC ASCVD risk.

Figure 2 shows the correlation between the LS values distributed into quartiles and the ASCVD REP system of four risk categories. Moreover, although the correlation of both is linear (r = 0.95), and the first two quartiles of the LS values coincide with the low and borderline ASCVD risk categories, the US score grossly underestimates the intermediate and highest risk categories (Q3 and Q4).

**Figure 3:**
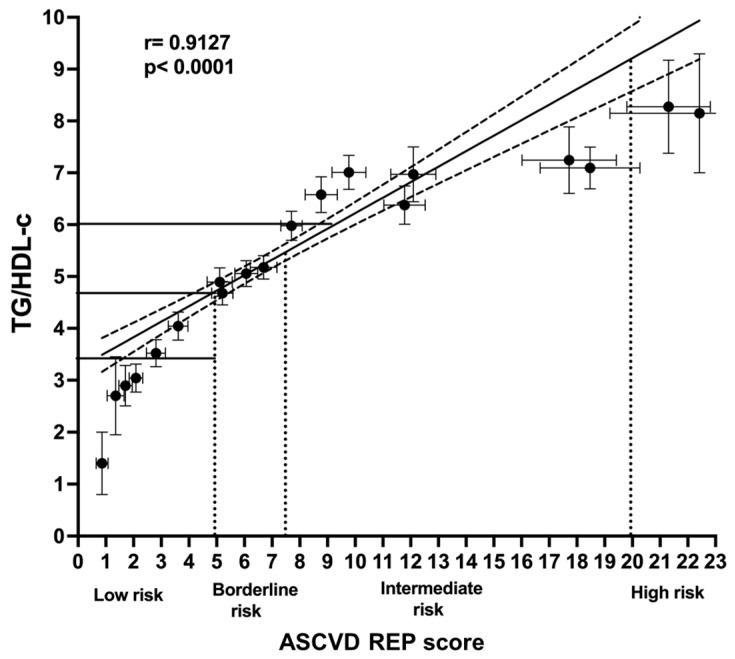
ASCVD REP score correlation with TH/HDL index. Solid horizontal lines correspond to TG/HDL-c risk groups, while dotted perpendicular lines express the four classical ASCVD REP risk groups. Although both variables are linearly correlated and fit correctly into the low-risk categories, the ACC ASCVD REP score grossly underestimates the highest risks.

A similar underestimation is observed in Figure 3, which shows the correlation between the TG/HDL-c index values and those of the ASCVD REP. Again, both systems had evident coincidences in the lowest-risk categories. However, the higher TG/HDL-c index values correspond only to the intermediate risk of the ASCVD REP scale.

## 4. Discussion

The Lindavista study population probably reflects contemporary Mexican inhabitants’ ominous anthropometric and cardiometabolic status [13,29,30], which explains the rise of the epidemic of cardiovascular diseases and diabetes that the country suffers. The epidemic of heart disease in Mexico causes more than 200,000 deaths yearly (with a rate of 178 per 100,000 inhabitants, 76% of which are due to ischemic heart disease) [4,31]. These diseases cause enormous expenses, not yet well quantified, that depend on in-hospital care, years of life lost, partial or permanent disability, and absenteeism from work. Many patients with acute coronary syndromes do not receive state-of-the-art treatment [32,33]. Therefore, Mexico has the most significant mortality due to myocardial infarction of all countries encompassed in the Organization for Economic Co-operation and Development (OECD) [34]. The lack of effective CV clinical assistance and prevention is aggravated by the chaotic situation in the health sector, fragmented into numerous federal and state institutions, some dedicated to the population under the protection of a social security system; others for the open population; and still others for particular segments, such as the armed forces, the oil workers, etc. Then, CV prevention in Mexico lacks leadership, infrastructure, health personnel, and long-term effective action plans.

Added to the generally poor, delayed, and backward therapeutic management of acute coronary syndromes, there is a lack of solid public preventive policies that can lighten the weight of the epidemic via primary and secondary prevention [35]. A population with a high prevalence of dysmetabolic O/O, sedentarism, HBP, and dyslipidemia is especially prone to developing ASCVDs [36]. Among the many tasks improperly performed in the field of prevention, we do not have a proven risk-scoring system that considers the distinctive characteristics of the Mexican population to establish preventive measures according to our national idiosyncrasy appropriately. The hypothesis backing this work is that risk scales and therapeutic recommendations must consider the traits secondary to specific ethnic, dietary, anthropometric, socioeconomic, and cultural determinants of every human community or nation. Human society is not homogeneous, and the assumption that a handful of risk factors have the same weight for the risk of myocardial infarction in all communities is simply untenable [37]. For example, a considerable segment of the Mexican population, mainly the urban low and middle classes, have combined dyslipidemia (hypertriglyceridemia and hypoalphalipoproteinemia) and increased LDL-c (lipid triad or atherogenic dyslipidemia) [9,13,33,36]. This lipid abnormality is typically observed in O/O and diabetic patients, in whom the binomial insulin resistance/hyperinsulinism syndrome underlies as a relevant physiopathological phenomenon and atherosclerotic risk factor [38]. As most risk scales do not include abdominal obesity, hypertriglyceridemia, and the lipid triad in their risk estimate, it is necessary to have a system that considers all of these typical traits of Mexicans to assess the CV risk adequately. There is robust evidence that abdominal obesity (increased abdominal girth), even with normal weight, is a powerful determinant of CV risk and recurrence of vascular events. As it is known, abdominal adiposity is strongly associated with insulin resistance; lipid abnormalities; and systemic inflammation, precursors, triggers, and aggravators of ASCVD [39,40,41].

The ASCVD REP score was chosen because it is the most accepted scale by Mexican physicians. The GLOBORISK [42] charts were ruled out because they are a scoring system that considers only six risk factors: age, gender, smoking status, diabetes, SBP, and TC. They cannot fully encompass the complex risk situation of a population seriously affected by O/O, insulin resistance, and atherogenic dyslipidemia. Furthermore, compared with the LS score or TG/HDL-c ratio, the results are more discordant than those seen with the ASCVD REP system. The latter score was made from the database of USA cohorts composed of White and Afro-American people of both genders [30,31]. Latin Americans (wrongly named in the US as “Hispanics” [43]) are not included in the pooled cohorts, even though this heterogeneous group represents the most significant minority proportion of the United States’ population [44]. In addition, it has been signaled that the ASCVD REP score may underestimate the accurate profile of some high-risk persons or overestimate risk in low-risk individuals [45].

We estimate the risk using the atherogenic ratio TG/HDL-c [21]. In comparison with the two other Castelli simpler risk quotients [23,46] (CT/HDL and LDL-c/HDL-c), where the numerator signals atherogenicity and denominator anti-atherosclerotic protection, the index TG/HDL-c expresses a more complex phenomenon, as it is associated with insulin resistance [47], atherogenic dyslipidemia [48], and the increment of small and dense atherogenic LDL [49] and VLDL particles. The index has been associated with the risk of myocardial infarction and ischemic heart disease [24,50,51], prediabetes development glycemic control in diabetic patients and mortalit [52,53,54,55], total mortality in women with suspected myocardial ischemia [56], the extension of coronary artery disease [57], arterial stiffness in apparently healthy persons [58], the incidence of intracranial atherosclerosis in non-stroke patients [59], high CV risk in obese children [60], cardiometabolic risks in women with the appropriate weight [61], and the metabolic syndrome [62,63], among many other pathological conditions.

We categorized the LS values in two ways: comparing the quartile values with those of the TG/HDL index and the ACC score estimate for ASCVD risk. A clearcut, straight correlation was observed between the TG/HDL index and the LS summation. However, it is necessary to point out that the cutoffs of the TG/HDL ratio have not been universally established, as the index varies significantly according to gender, body mass index, insulin resistance, ethnicity, comorbidities, and other determinants [64,65,66,67,68]. Despite certain correspondence between both scales, intermediate-risk categories had considerable overlap. We determined the index values related to the quartile distribution (Figure 1). Then, we compared these percentile-defined LS categories with the established ACC ASCVD REP categories (Figure 2), observing that the US system, despite the straight linear correlation, underestimates the risk assessed, as shown by the LS summation. The same inconsistency was found between the ASCVD REP and the TG/HDL-c, using the index values derived from the quartile LS distribution. Any scale that does not include TG, low HDL-c, atherogenic dyslipidemia, or TG/HDL-c cannot represent the complex metabolic situation that assails the contemporary Mexican population.

An essential fact about the TG/HDL ratio is that it is a marker of atherogenic dyslipidemia. The atherogenic power of this lipid complex was well established in the Münster study, in which, although just 4.3% of the cohort population was affected by atherogenic dyslipidemia, this small proportion of patients concentrated 25% of the coronary events [69].

This simple risk index has been proven to be a useful clinical tool in a handful of Mexican studies published in national and international journals by different research groups [9,61,62,70,71,72].

## 5. Conclusions

This study shows that the TG/HDL-c index portrays the risk faced by a population havocked by abdominal obesity and diabetes better than the US ASCVD REP score system. We developed a risk score system that reflects the particularities of the contemporary Mexican population. One of the study’s limitations is that the nonprobabilistic sample of the Lindavista study (formed by urban middle-class inhabitants of Mexico City) represents only part of the Mexican population. As it is known, Mexico has notable regional differences from the ethnic, economic, educational, nutritional, and anthropometric points of view. The high correlations of the TG/HDL-c quotient with the ASCVD REP score and the proposed Lindavista score signal that this index is a valuable, simple, and inexpensive tool to assess cardiovascular risk. With the need to prospectively test the Lindavista score in a more representative and broader sample, the TG/HDL ratio is the only current measurement that can accurately indicate the CV risk of Mexican patients.

What should follow these observations is to test the usefulness and certainty of both the Lindavista score and the TG/HDL-c ratio in a study whose sample represents all the regions of the country to prove their capacity to predict cardiovascular outcomes.

## Figures and Tables

**Table 1:** Lindavista cardiovascular risk score.

Risk Factor Grading	Scoring
Age (years). Female	
<30	−3
30–39	−1
40–49	0
50–59	1
>60	2
Age (years). Male	
<30	−1
30–39	0
40–49	1
50–59	2
>60	3
Smoking (daily consumption)	
Never smokers or former smokers (at least in the last year)	0
Cigarette consumption	
1–5 per day	1
6–10 per day	2
>10 per day	3
Body mass index (kg/m^2^)	
<25	0
25–29.9	1
30–34.9	2
≥35	3
Abdominal circumference in women (cm)	
<80	0
80–84.9	1
85–89.9	2
≥90	3
Abdominal circumference in men (cm)	
<90 cm	0
90–94.9 cm	1
95–99.9 cm	2
≥100 cm	3
Systemic systolic blood pressure (mm Hg)	
<140	0
140–159	1
160–179	2
≥180	3
Systemic diastolic blood pressure (mm Hg)	
<90	0
90–99	1
100–109	2
≥110	3
Fasting glycemia (mg/dL)	
<100	0
100–126	1
127–140	2
≥140	3
Total cholesterol (mg/dL)	
<200	0
200–239	1
240–279	2
≥280	3
Triglycerides (mg/dL)	
<150	0
150–199	1
200–499	2
≥500	3
HDL-c (mg/dL)	
≥60	0
40–59	1
30–39	2
<30	3
LDL-c (mg/dL)	
<100	0
100–129	1
130–159	2
≥160	3

The table shows all factors considered to obtain a global summation of risk. Their sum expresses the number and severity of adverse determinants of CV risk. The gradation of each one was performed in some cases by the universal acceptance of standard categories, for example, the cholesterol or triglycerides concentration categories or the grades of dysglycemia. In other cases, the category breakdown was arbitrary (for example, abdominal obesity). Acronyms are defined in the text.

## Data Availability

Data are available upon request.
